# Ab-Initio Spectroscopic Characterization of Melem-Based Graphitic Carbon Nitride Polymorphs

**DOI:** 10.3390/nano11071863

**Published:** 2021-07-20

**Authors:** Aldo Ugolotti, Cristiana Di Valentin

**Affiliations:** Dipartimento di Scienza dei Materiali, Università degli Studi di Milano-Bicocca, Via Cozzi 55, 20125 Milano, Italy

**Keywords:** graphitic carbon nitride, melon, melem polymerization, XPS, NEXAFS, XRD, DFT, surface science

## Abstract

Polymeric graphitic carbon nitride (gCN) compounds are promising materials in photoactivated electrocatalysis thanks to their peculiar structure of periodically spaced voids exposing reactive pyridinic N atoms. These are excellent sites for the adsorption of isolated transition metal atoms or small clusters that can highly enhance the catalytic properties. However, several polymorphs of gCN can be obtained during synthesis, differing for their structural and electronic properties that ultimately drive their potential as catalysts. The accurate characterization of the obtained material is critical for the correct rationalization of the catalytic results; however, an unambiguous experimental identification of the actual polymer is challenging, especially without any reference spectroscopic features for the assignment. In this work, we optimized several models of melem-based gCN, taking into account different degrees of polymerization and arrangement of the monomers, and we present a thorough computational characterization of their simulated XRD, XPS, and NEXAFS spectroscopic properties, based on state-of-the-art density functional theory calculations. Through this detailed study, we could identify the peculiar fingerprints of each model and correlate them with its structural and/or electronic properties. Theoretical predictions were compared with the experimental data whenever they were available.

## 1. Introduction

Among carbon–nitrogen compounds, polymeric carbon nitrides have attracted much attention during the last few years, especially in the field of photocatalysis [1,2,3,4,5,6,7,8,9], because of their long-term stability, nontoxicity, cheapness, and electronic structure, suitable for the activation of a large number of photochemical reactions of interest for applications in the green economy [10,11,12,13]. First, the reactivity of carbon nitride polymers is driven by their peculiar structure, made of regularly spaced voids, exposing pyridinic N atoms, which improve the catalytic activity of N-decorated graphene [14,15,16] and are important coordination centers for transition metal atoms, in analogy to N-rich macrocycles, such as porphyrins [1,17,18]. Second, under standard conditions, these materials are most stable in layered structures [19,20], in a graphite-like fashion, commonly called graphitic carbon nitride (gCN), which can be further processed in order to exfoliate single two-dimensional sheets [3], exposing larger surface areas than those of the stacked structures and being more suited to host chemical reactions. Additionally, the synthesis of gCN can be achieved through a large number of pathways, exploiting different precursors and different reaction conditions [1,7,9,11]: most notably, in several cases, the material can be grown in dry environments without the presence of a supporting surface. In one of the most common processes, a molecular precursor, i.e., melamine (2,4,6-triamino-s-triazine or C${}_{3}$N${}_{6}$H${}_{6}$), is taken in the form of a powder that is heated at different temperatures to allow for its condensation into melem monomers first (2,4,6-triamino-tri-s-triazine, C${}_{6}$N${}_{10}$H${}_{6}$), and then polymerization into a stacked two-dimensional network [2,3].

In addition to its aforementioned pristine properties, such a compound has more recently been studied as an ideal platform to host further functionalizing species that improve the chemical activity of gCN: among these, the inclusion of transition metal atoms is one of the most exploited. For example, the inclusion of isolated Fe atoms was reported to improve the α-cyanation of secondary and tertiary amines [21], whereas Cu adatoms were found to benefit the reduction of CO${}_{2}$ [22,23] or in combination with Ni adatoms, to mediate the methanol electro-oxidation [24].

However, despite the apparent simplicity and tunability of the synthesis of gCN described above, the final product of the process is far from being unique or uniform: partial condensation or polymerization can lead to the formation of polymorphs with largely different structures. In particular, even assuming a complete pyrolysis of melamine into melem, the latter can polymerize into several configurations [4], depending on the number of the NH${}_{2}$ functional groups removed from the monomer, and thereby on the resulting stoichiometry between C and N: the most famous ones are (i) Liebig’s melon with a C/N ratio of 2:3, and (ii) the ideal fully polymerized gCN (igCN) with a ratio of 3:4. The correct identification of the obtained gCN polymorph is a key preliminary step when studying the catalytic performances of the prepared samples. Nonetheless, from an experimental point of view, several ambiguities arise in the characterization of gCN samples, the first being the difficulty to directly acquire images of the surface at the nanoscale (for instance by atomically resolved microscopies), either because growth does not take place on a support or because of the presence of a liquid environment.

One of the most common techniques exploited for investigating the sample structure with atomic-scale resolution is X-ray diffraction (XRD), whose spectral interpretation is largely influenced by the choice of the reference model: for example, the strongest spectral feature is related to the distance between stacked planes and is not very sensitive to the actual in-plane atomic structure. If the chemical composition is probed, instead, similar complications arise: gCN samples are commonly studied through X-ray photoelectron spectroscopy (XPS) whose experimental interpretation is usually performed by direct comparison with known and supposedly similar systems.

From a computational perspective, the majority of theoretical studies present in the literature are mainly focused on the investigation of the atomic structure, whereas in some cases, the electronic properties are also discussed, with particular reference to the optical band gap, and only in very few cases the simulation and analysis of the X-ray spectroscopies are presented [25]. However, not all geometric degrees of freedom are considered in those works, such as the proper size of the supercell for igCN or the relative arrangement of the monomers for melon: most frequently, the layer is assumed to be perfectly flat [10,22,26] as performed for experimental characterization, and only Liebig’s configuration for a melon is considered [27,28], respectively. Such biased approaches have already been questioned by others, who, for example, studied corrugated igCN models [29,30], discussed melon formation rather than igCN [27,28], or proposed an alternative structure for melon [23]. All of these works suggest that detailed evidence from the prepared samples under study must be carefully reviewed in order to correctly identify the structure of the synthesized polymorph present in the sample. However, a comprehensive comparison of structural and spectroscopic properties of different gCN models is still missing.

For these reasons, in this work we investigate, by means of density functional theory calculations, several polymorphs of melem-derived gCN to achieve a full characterization of the structure, the electronic and spectroscopic properties. In particular, we simulate the XRD, XPS and near-edge absorption fine-structure (NEXAFS) spectra and unambiguously identify the differences observed for the various models. We also study the effect of relieving constraints from a priori assumptions about the structure of gCN polymorphs on the resulting spectral features, as we consider partial polymerization, complete exfoliation, or layer corrugation.

## 2. Computational Methods

We carried out all simulations through the density functional theory (DFT) framework using the QuantumESPRESSO suite [31,32,33]. We employed ultrasoft pseudopotentials taken from the PSlibray [34], with a plane-wave basis size of 46 Ry for the wave function, and 326 Ry for the charge density. We relied on the PBE exchange-correlation functional [35] and included the dispersion forces through the Grimme-D3 scheme [36]. For selected systems, we used the coordinates optimized with a PBE functional to run single-point calculations using the hybrid B3LYP functional [37]: in those cases, we used norm-conserving pseudopotentials derived from those included in the SG15 library [38,39], with 70 Ry cutoff for the calculation of both wave functions and exact exchange operator. For those systems with periodic boundary conditions, we ensured that the size of the supercells was large enough to allow for the proper structural degrees of freedom during the optimization, i.e., by including four monomers. In the case of two-dimensional structures, we included a vacuum region of at least 13 Å along the non-periodic direction, which minimizes the interactions between a layer and its replicas. For the self-consistent calculation of the wave functions, we considered a shifted 2 × 2 × 1, 2 × 2 × 2 or 2 × 2 × 6 grid of k-points, generated through the Monkhorst–Pack method [40], for 2D melon/igCN, bulk igCN, and bulk melon models, respectively. Such a grid was multiplied by a factor of 3 along each periodic direction for the calculation of the density of states (DOS).

XRD spectra were constructed by applying a Gaussian broadening of 0.2${}^{\circ }$ to the structure factors calculated through the Fourier transform of the three-dimensional charge density as performed by VESTA software [41,42]. For the spectra of two-dimensional structures, where the artificial periodicity required by the plane-waves basis would generate spurious diffraction peaks, only those reflection planes with Miller index along the non-periodic direction equal to 0 were included.

The binding energies (BEs) of the inequivalent N atoms at the K-edge were evaluated up to an unknown constant through the ΔSCF method [43]. This approach requires to run single-point calculations including, for each target atom, a PAW pseudopotential [44] modified in order to host a core hole in the 1*s* state. As soon as an energy reference is chosen, for example, the averaged total energy of pyridinic N atoms, it is possible to calculate the core level shift (CLS). Additionally, we convoluted the CLSs with Gaussian functions, with broadening σ = 0.17 eV, for the construction of the XPS spectra. In order to compare the CLS of different two-dimensional supercells and their chemical shift, we included an N${}_{2}$ molecule sufficiently far from the surface, acting as a common reference: such an approach was proven to be robust in previous works of our group [23,45]. To carry out the calculation of the chemical shift for bulk systems, we optimized slabs with 3 layers, keeping the lowest two fixed, calculated the XPS spectra including the N${}_{2}$ reference, and only considering the CLSs of the intermediate layer.

To construct the NEXAFS spectra, we relied on the transition-potential method [46], which requires the use of half-core hole pseudopotentials; then, the absorption matrix elements were calculated using the XSpectra tool included in QuantumESPRESSO [47]. The final NEXAFS spectra were obtained with a uniform broadening of 0.2 eV.

## 3. Results and Discussion

### 3.1. Structural Properties by Simulated XRD Characterization

**Figure 1:**
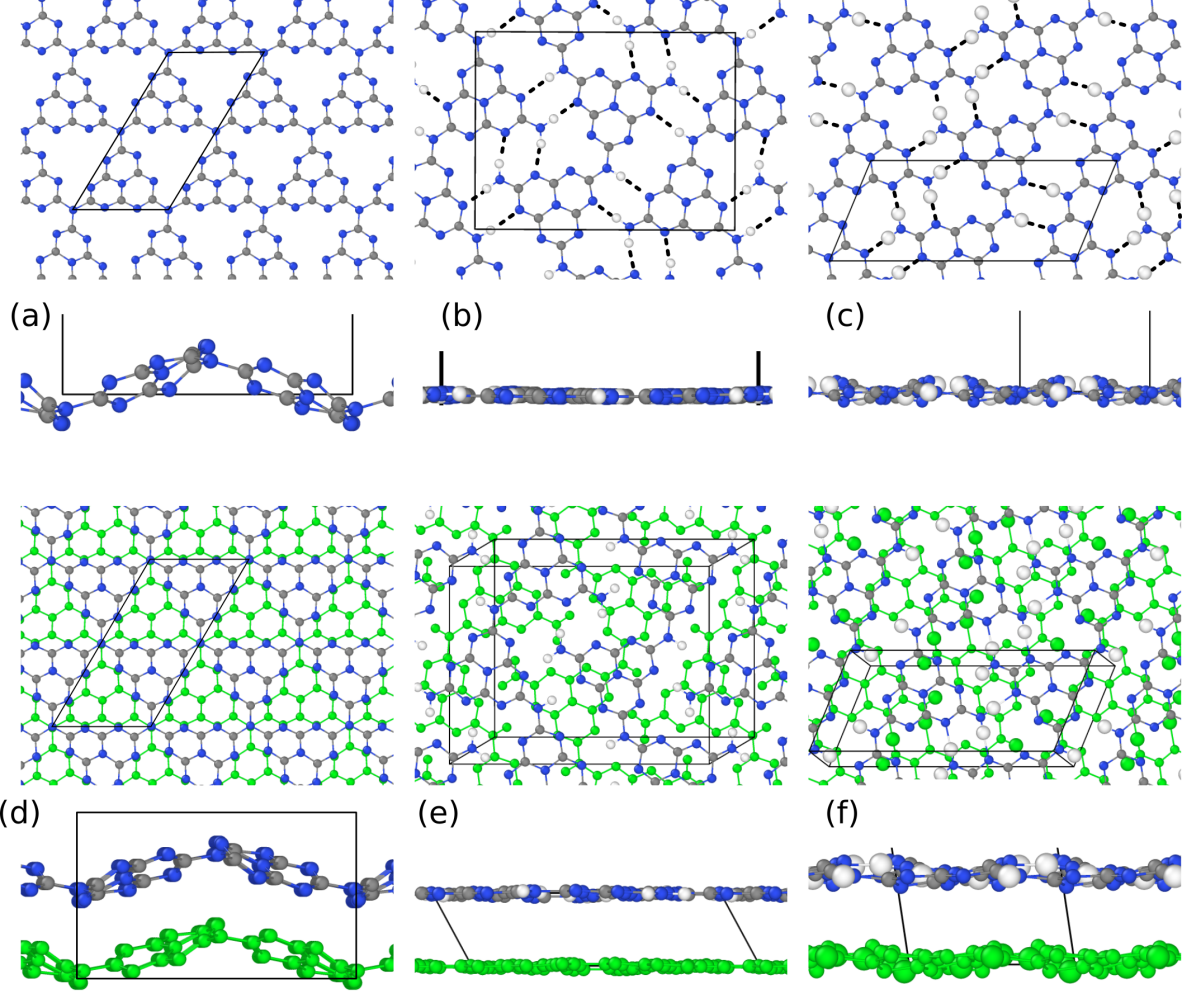
Top and left side views (with different scale) of optimized structures of different gCN models: (**a**,**d**) igCN, (**b**,**e**) melon alternated, (**c**,**f**) melon linear. Top panels report 2D structures; lower panels, corresponding bulk geometries. The unit cell structure is shown with black lines. Blue, gray, and white spheres represent N, C, and H atoms, respectively. For bulk systems, the second layer included in the supercell is shown through green spheres. For the melon models, the H-bonds are reported only in the 2D case with dashed lines.

We first present the structure of two-dimensional (2D) single layers of fully and partially polymerized gCN polymorphs, namely, igCN and melon, respectively, which are reported in Figure 1a–c. Full polymerization involves the conversion of all three primary amines in a melem residue into tertiary amines, whereas partial polymerization in melon limits the conversion of two primary amines into secondary ones. The corresponding 3D layered structures are also considered for comparison. These bulk models, shown in Figure 1d–f, include one or two layers in the simulation cell accounting for different arrangements of the stacked planes.

We propose two melon models, where the monomer polymerizes according to different patterns, linear or alternated, as shown in Figure 1b,e and Figure 1c,f, respectively. The 2D igCN sheet was highly corrugated, with buckling up to 3.0 Å, whereas the linear melon layer had a buckling of 1.0 Å and the alternated one was almost flat. Such distortions were slightly modified in the corresponding bulk models, 2.6 and 1.4 Å, due to the presence of the additional layers: the different behavior of igCN with respect to the melon linear model was due to the different stacking configuration, namely, the AB and AA types, respectively. In the case of igCN, both AA and AB stacking configurations could be built, but the AB one, involving the rotation of one layer by π/2 around the normal direction and its in-plane translation, was more favorable by 1.4 eV. In the case of the melon models, instead, the only possible staking configuration was AA, since only in-plane translation was permitted. Our optimized structures, shown in Figure 1, agreed with those already reported in the literature for igCN [13,29,30,48] (in particular, in the last two references, a rather smaller corrugation was calculated) and alternated melon, where, however, no corrugation [28] except an artificial sinusoidal one [49] was reported.

**Figure 2:**
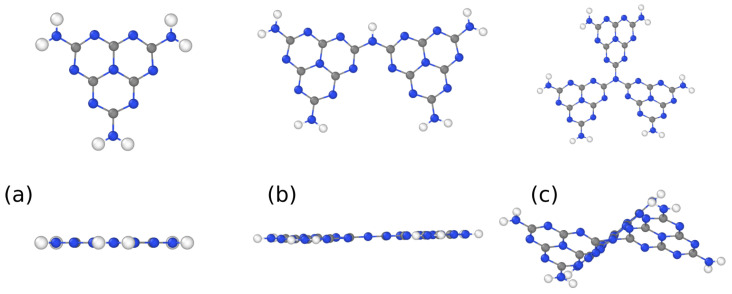
Top and front views of the optimized structures of melem (**a**) monomer, (**b**) dimer and (**c**) trimer. Blue, gray, white spheres represent N, C, H atoms respectively.

The corrugation of gCN is driven by the short-range electronic repulsion between pyridinic N atoms. To verify the role of N lone pairs at the monomer borders, we investigated three molecular species: the melem monomer, the dimer, and the trimer, whose optimized structures are shown in Figure 2. The dimer was still quite flat with a little in-plane distortion that minimized the repulsive interaction; in the trimer, however, the central N joint is a constraint that forces each monomer to twist around the threefold C${}_{2}$ axes passing through it.

Indeed, such a repulsive effect is modulated in the melon models by the formation of stabilizing H bonds with an average length of 2.0–2.2 Å, between pyridinic N atoms and the amino groups belonging to different rows of melem polymers. The actual position of the H-bonds created by primary or secondary amines is driven by the different arrangement of the monomers, which may affect the reactivity of the two systems (see Figure 1).

Even though dispersion forces are not the major source of the distortion in gCN, we assessed the possible impact that a different choice of the method used to include van der Waals interactions would have had on the structural parameters and atomic positions. To this aim, the structure of 2D and bulk igCN was reoptimized using the non-local VDW-DF2-C09 exchange-correlation functional [50,51,52,53], and results were compared with those with Grimme-D3 used in the present study. The maximal and average differences in the atomic positions were less than 0.06 Å for both 2D and bulk igCN. In the case of the lattice parameters, the most influenced one was the stacking distance between planes in the bulk model, with a difference of 4%, while the others (in-plane lattice distances and angles) varied by much less than 1% both in the bulk and 2D models.

**Figure 3:**
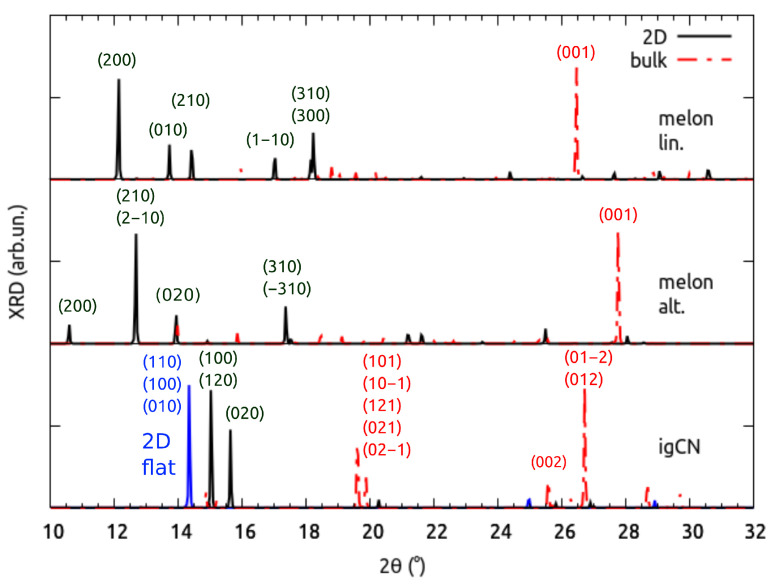
Simulated XRD spectra of optimized gCN models: intensities are scaled in order to match the height of the most intense features. The strongest peaks are labeled with the indices of reflection planes.

In the following, we present the simulated XRD characterization of the above models, whose spectra are shown in Figure 3. In the case of bulk models, the linear melon presents the main reflection at 26.4${}^{\circ }$, which was notably different from that of the alternated one at 27.7${}^{\circ }$, making the two models easily distinguishable through an experimental XRD measurement. Both these peaks were due to the reflection between stacked layers. In the case of bulk igCN, instead, the main reflection peak was located at 26.7${}^{\circ }$ and was not attributed to the (002) plane, as one would expect for a highly symmetric structure (see below), but rather to the (012) one, which we deemed to be related to the highly buckled geometry. Additional features in the spectrum allow for distinguishing the fully polymerized structure from the others: a set of peaks around 19.7${}^{\circ }$, which were due to the reflections from planes with higher Miller indices. The peaks corresponding to the in-plane periodicity were not absent, but were so weak that they could not be resolved in our simulated spectra of bulk models.

Despite the relative sensibility of the stacking distance (4%) to the computational setup, i.e., using the Grimme-D3 correction or the non-local VDW-DF2-C09 functional, as discussed above, the effect on the position on the high-angle peak was smaller than 1${}^{\circ }$ for all models under investigation, with no consequences on the considerations above.

When analyzing the 2D structures, we discarded any reflection plane not parallel to the *xy* one, which certainly did not appear in the case of exfoliated sheets. In the resulting XRD characterization, the two melon models were less clearly identifiable than the bulk case was, as the most intense peak in the spectrum of linear melon, observed at 12.1${}^{\circ }$, was very close to that of alternated melon at 12.7${}^{\circ }$. However, they could be addressed to different reflection planes, i.e., (200) or (210)/(2$\bar{1}$0) whose representation is given in Figure S1. The full set of optimized crystal parameters in reported in Table S1 in the Supplementary Materials.

In particular, the alternated melon could be identified through the less intense peak occurring at 10.6${}^{\circ }$, originated by (200) planes, which did not have a correspondence in the spectrum of linear melon due to their different crystal structures. The 2D igCN could be very clearly distinguished because its XRD spectrum showed two intense peaks at much higher angles (15.0${}^{\circ }$ and 15.6${}^{\circ }$) than in the spectra of the two melon models.

Next, to extrapolate the effect of corrugation, we constructed a model of 2D igCN where the atoms had been fixed in the same *xy* plane during the atomic relaxation, as shown in Figure S2a. In the corresponding XRD spectrum, reported together with that of the fully relaxed corrugated model in Figure 3, is it possible to observe a single peak (not two) at 14.3${}^{\circ }$, which was equally originated by the reflections from the (100), (010) and (110) planes, given the highly symmetric threefold crystal structure.

We conclude this section with some comparison with existing studies. The simulated XRD features for alternated melon observed in the present work were in good agreement with those previously calculated and available in the literature [27,49]. For igCN, however, we must make some remarks. First, we assigned the reflection plane for the most intense peak to (012) instead of (002), which is expected when assuming a regular flat system. When comparing the position of this calculated line with those proposed in previous experimental works, we observed better agreement when repeating cell models with a larger lattice parameter [27] rather than with a smaller one [49]. Similar comparative analysis also held for the position of the (100) peak at ∼14–16${}^{\circ }$. Second, we observed the presence of several peaks related to higher Miller index planes, such as the (120) and the (020), which were not considered in previous works [27,49].

The direct comparison with experimental spectra, where major differences were in the position of XRD peaks at low angles, suggests that, depending on the growth conditions during synthesis, one can obtain either a homogeneous melon [27,28,49,54], a mixture of the two melon structures [23], or igCN [24] structures.

### 3.2. Electronic Properties by Simulated X-ray Spectroscopies

**Figure 4:**
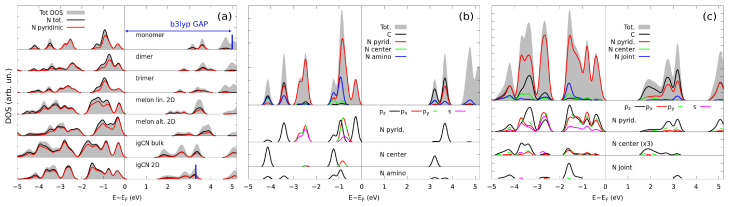
Total density of states and its projections onto selected atomic orbitals. (**a**) Comparison among different gCN systems and precursors. Blue vertical lines mark the position of the bottom of the conduction band calculated with the B3LYP functional. (**b**,**c**) Comparison of PDOS among C and different types of N atoms in monomer and 2D igCN, respectively.

In this section, we discuss the electronic characterization of igCN and melon, as obtained through simulated X-ray spectroscopies. First, we compare the ground-state electronic structure, including that calculated for monomers, dimers, and trimers, in order to describe the changes caused by a different degrees of polymerization. The total DOS and its projections onto selected atomic orbitals (PDOS) calculated for the different models are shown in Figure 4a.

Moving down along Figure 4a, the top of the valence band was exclusively composed of N states, most of which from the pyridinic atoms, while the bottom of the conduction band was composed of hybrid C-N states. As polymerization proceeded, these bands just became thicker but did not change their character, independently from the type of the functional group or link between the monomers, namely, primary, secondary, amines, or threefold coordinated N. The HOMO-LUMO gap steadily decreased from 3.5 eV in melem to 2.55/2.34 eV (direct) in alternated/linear melon to 1.80 eV (direct) of either bulk or 2D igCN. Since the PBE functional underestimates the energy gap of semiconductors, we performed single-point calculations using the hybrid B3LYP functional of the monomer and of 2D igCN in order to improve the accuracy of our results: in this case, the resulting HOMO-LUMO gaps were 5.01 and 3.22 eV, respectively, much larger than the experimentally reported value of 2.7 eV [55,56]. Compared to other theoretical results, however, the intrinsic band gap seemed extremely sensitive towards the numerical setup, as our value was in good agreement with that previously reported for a melon [28] but less for igCN [13,48], as different corrugations were obtained [29] or other hybrid functionals [30,57] were taken into account, the latter option resulting in band-gap values closer to the experimental value. As a further step, we carried out a deeper analysis of the PDOS on N atoms for the melem monomer and 2D igCN, reported in Figure 4b,c, where we distinguished among (i) the pyridinic N, the different types of three-fold coordinated N, i.e., (ii) the N atoms at the center of monomers (graphitic), (iii) those at the joint and (iv) the N atoms within amino groups (primary and secondary). In the case of the monomer, we first focus on the pyridinic N atoms. The contribution of the in-plane *p* orbitals overlapped that of the *s* orbital at −0.8 and −2.5 eV; therefore, we attributed those states to the lone pairs. The projections of the $p_{z}$ orbital were more distributed along the energy range; especially at −3.5 and −5.3 eV, they overlapped with the projections on C states; therefore, we attributed these contributions to the formation of the delocalized Π band. Focusing instead on the three-coordinated N atoms, the major PDOS contributions in the selected energy range were those by the $p_{z}$ orbitals, which were rather scattered, appearing at around −1.25 eV and between −3.5 and −4.5 eV. This suggests some hybridization with $p_{z}$ C states. The center N atoms and the amino N ones, however, had a different behavior: the former showed a much larger overlap with the projections on C states, especially at lower energies, which is an indication of the formation of delocalized Π bonds. Conversely, amino N atoms retain electronic charge on their $p_{z}$ orbitals to a larger extent. Similar analysis could be carried out in the case of 2D igCN, with the additional complication given by the relative mixing of the contributions from the three *p* states caused by the corrugation of the layer, which is particularly evident for pyridinic and graphitic N atoms. A tentative sketch of the electronic configuration of a monomer in 2D igCN is shown in Figure S3.

**Figure 5:**
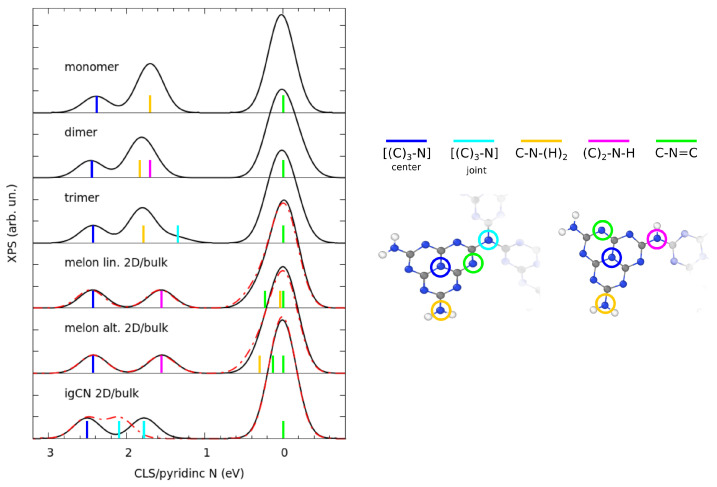
Comparison of XPS spectra calculated for different models of gCN and melem aggregates. Solid black lines or dash-dot red lines report spectra of 2D or bulk structures, when available. CLSs calculated aligning the position of pyridinic N atoms to the origin. The position of the contributions from inequivalent N atoms is marked on the spectra through colored sticks.

We now present the chemical characterization of gCN as obtained through the simulation of the XPS spectra at the N K-edge. Given our computational setup, we focus on the relative energy separation between different spectral features, which are highlighted in Figure 5 and aligned with reference to the pyridinic N peak. In all considered models, independently from the level of polymerization, the N atoms at the center of the monomers had the highest BE, with an almost constant CLS of 2.5–2.6 eV. This result was expected, since the BE (and consequently the CLS) is inversely proportional to the electronic charge localized around a specific atom; therefore, a pyridinic N shows a lower CLS than a graphitic one due to the different behavior of the lone pair discussed above. The assignment given in Figure 5 is in contrast with several reported works, where the amino and graphitic components are systematically inverted [1,20,54,58,59,60,61,62]. However, our analysis is supported by that of other studies [28,63] that also relied on theoretical calculations. Moreover, our approach is rather robust since we selectively targeted each different N type atom in the models, and because the same trends were confirmed by the parallel study on the melem monomers, dimers, and trimers.

Our calculated spectra present an additional feature at CLS of ∼1.6–1.8 eV, which was due to tertiary, binary, or primary amines, depending on the considered model. In two-dimensional structures, for example, the fully polymerized model presented threefold coordinated N atoms joining the monomers, while in melon models, the joints were composed of secondary amines; conversely, for melem aggregates (dimers and trimers), this spectral feature is only originated by amines. Interestingly, the primary amino component in both a linear and an alternated melon is shifted towards a much lower CLS due to the formation of H-bonds with the pyridinic sites located on the polymeric chain nearby. As a consequence, an additional two-coordinated component could be identified at a slightly positive CLS. Such an effect of H-bonding was reported in the case of melamine-based gCN [25]. Comparing 2D with 3D structures, we observed clear effects on the simulated XPS spectra, when the stacked sheets in the supercell had a different relative arrangement. In the case of melon models, since the stacking order followed an AA pattern, no effect on CLS was observed. On the other hand, for igCN, the peculiar AB pattern allowed for an overlap of the atomic orbitals from atoms of different layers at the joint sites, which could be the reason for the blueshift of the CLS of such component.

The comparison with the experimental results available in the literature is indeed made complicated by the uncertainty about the actual structure present in the samples. Our results for melem and for the alternated melon model, however, are in good agreement with those reported in [28], both for the position of the peaks and their relative intensity. More generally, discrepancies that one can observe from our predictions involve the position, but more frequently the intensity, of the features contained in the XPS spectra [1,20,28,54,58,59,60,61,62,63]. This could be due to the formation of not-so-pure patches of gCN, but also to the influence of the growth environment, namely, the presence of a supporting surface. Regarding the synthesis of a non-uniform sample, in a recent work by our group about the characterization of Cu-functionalized gCN [23], we suggested that the real sample was composed of a mixture of gCN polymorphs, since the experimental XPS spectrum resembled a sum of the simulated XPS spectra for linear and alternated melon models. In order to give additional insight to this hypothesis, we calculated, for each model, the chemical shift with respect to a common reference, namely, an isolated N${}_{2}$ molecule inserted into the simulation cell, as reported in Table 1. Additionally, in Figure S4a, we present the comparison between XPS spectra, including the calculated chemical shifts.

These values suggest a model-dependent degree of charge delocalization on the Π system, which increases with the level of polymerization. The chemical shifts of bulk structures were estimated from a slab calculation and assumed to be equal. Such an assumption can be validated a-posteriori by comparing the slab and bulk XPS spectra. In the case of the melon models, they were identical. In the case of igCN, they were not. However, the internal layer of the slab behaved as one of the bulk; thus, we could take it as our reference, as shown in Figure S4b. To complete our characterization, when constraining the igCN model to be flat, its chemical shift would be 0.4 eV with respect to the common reference (see the simulated XPS spectrum in Figure S4c).

**Figure 6:**
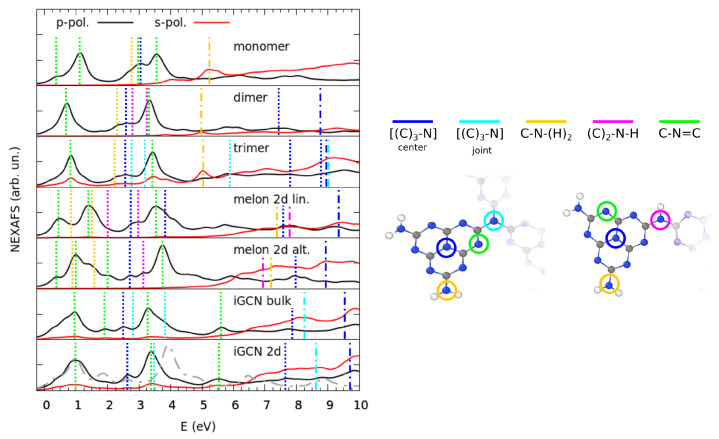
Comparison of NEXAFS spectra calculated for different models of gCN and melem aggregates for both X-rays polarizations. Excitation energies were shifted aligning the position of the onset of the absorption. For 2D igCN, the gray dot-dash line shows the spectrum of the flat model with p polarization. The position of the contributions from inequivalent N atoms for the p or s polarization is marked on the spectra through the vertical dashed or dot-dashed lines, respectively.

Lastly, we present further details of the electronic structure from the simulation of the NEXAFS spectra at the N K-edge, which are shown in Figure 6, together with the contributions of the different N species. In all models, the out-of-plane polarization showed strong absorption at much lower energies than the in-plane one. Such dichroism is expected for planar structures; indeed, in the case of the trimer and 2D igCN, the cross-section of the s-polarization was non-negligible in the low-energy range, which is a reflection of the distortion of the geometry due to the rotation of the monomers or the buckling, respectively.

Taking the spectrum of the melem monomer as a reference system for our analysis, the fingerprints of the electronic structure discussed above were observed: for the p-polarized spectra, there were two main features at ∼1.0 and 3.4 eV, which were related to the pyridinic $p_{z}$ electrons involved in the $\Pi^{*}$ states. The bumps in the spectra at 2.7–3.0 eV could be assigned, instead, to the antibonding states involving the hybrid C-N $p_{z}$ orbitals, mostly belonging to the three-coordinated N atoms at the center of monomer triangles, but also in part to amino groups. These states lie in the same energy range than the pyridinic ones in the PDOS (Figure 4), but the resonant excitation process represented by the NEXAFS spectrum also includes the energy cost of generating the core hole, which was calculated for the XPS simulation above, and explains the blueshifted position of the contribution of the three-coordinated N atoms with respect to the (first) pyridinic peak at low energy. In the s-polarized spectra, the major contributions could be observed at higher energies, for example at 5.2 and ∼7.5 eV, which were related to the σ states of amino N types. Given the stoichiometric ratio of the different N species, spectra were inevitably dominated by the pyridinic components. For a more detailed comparison, an atomically resolved NEXAFS characterization is shown in Figure S5. As expected, the spectrum of bulk igCN was very similar to that of the 2D model, aside from two differences: the presence of an additional pyridinic component at ∼2 eV, and the blueshift of 0.4 eV of the N joint component, caused by the interaction between stacked layers. When the constrained flat model was taken into account, the whole absorption was blueshifted, as expected from the increased electronic repulsion between pyridinic lone pairs within the plane.

The calculated structure of the NEXAFS spectra was in good agreement with those reported in the literature [56,64] for igCN; however, similarly to XPS characterization, we obtained a different attribution of the peaks. In particular, we assigned the second most intense feature, located at a photon energy of 3.2–3.5 eV (corresponding to, roughly, 402–404 eV in the experimental results), to the absorption by pyridinic N, while in the literature, it is often related to a graphitic N atom [56,64].

## 4. Conclusions

In this work, we built and optimized the atomic structure of several polymorphs of melem-based gCN, namely, the partially polymerized melon (with different arrangements of the monomer) and the fully polymerized igCN, both bulk and exfoliated 2D systems. Moreover, we considered for comparison the corresponding melem monomer, dimer, and trimer. For all these models, we investigated the structural and electronic properties through the simulation of several of the most common experimental techniques of characterization, namely, XRD, XPS, and NEXAFS. The first result of this theoretical characterization is the definition of the origin of the corrugation of the 2D sheets as due to the electrostatic repulsion of the pyridinic N sites. Second, through the comparison of the simulated XRD spectra and available experimental measurements, we identified key reflection planes that were found to be distinctive of each structure. Third, we determined the contribution of the different N species (pyridinic, graphitic center, joint, NH${}_{2}$, and NH) in the electronic structure (PDOS) of various polymorphs. In terms of simulated XPS data, the level of polymerization and the arrangement of the monomers left unique fingerprints in the structure of the spectra. A comparison with experimental results was indeed complex given the number of different growth conditions influencing the measurement. To take into account this issue, we discussed the attribution of the XPS features with reference to more possible polymeric structures that could be present even simultaneously in the same experimental sample. Lastly, we studied the simulated NEXAFS spectra, among which the ones for the fully polymerized models are in the best agreement with the experimental results reported in the literature. Our analysis clarified the attribution of the spectral features. 

## Figures and Tables

**Table 1:** List of chemical shifts (in eV) between XPS spectra calculated for different models of gCN polymers and melem aggregates with respect to a common reference, an isolated N${}_{2}$ molecule inserted into the simulation cell.

Model	Isolated	2D	Bulk
Monomer	−2.6	-	-
Dimer	−2.6	-	-
Trimer	−2.5	-	-
Melon linear	-	−1.4	−1.6
Melon alternated	-	−2.3	−3.1
igCN	-	−1.7	−1.7

## Data Availability

The data presented in this study are available on request from the corresponding author.

## Supplementary Materials

The following are available https://www.mdpi.com/article/10.3390/nano11071863/s1, Figure S1: representation of real space reflection planes associated with the main peaks reported in the XRD spectra of the 2D models, Table S1: list of the optimized crystal parameters of the optimized unit cell of the periodic models, Figure S2: top and left side view of the optimized structure of the alternative 2D gCN (flat) systems, Figure S3: sketch of a monomer of 2D igCN and its electronic structure in real space, Figure S4: comparison of simulated XPS spectra, including the relative chemical shift, of bulk/2D/slab igCN and of 2D igCN flat/distorted, Figure S5: detailed atomically resolved NEXAFS spectra for the different models.
