# Supplementary material for: Ab-Initio Spectroscopic Characterization of Melem-Based Graphitic Carbon Nitride Polymorphs
Source: Nanomaterials (Basel). 2021 Jul 20;11(7):1863. doi: 10.3390/nano11071863 (PMC8308387; doi:10.3390/nano11071863)
Supplement: Supplementary file 1 [file nanomaterials-11-01863-s001.zip › nanomaterials-1281282-supplementary.pdf]

Supplementary Materials: Ab-Initio Spectroscopic Characterization of Melem-Based Graphitic Carbon Nitride Polymorphs

Aldo Ugolotti and Cristiana Di Valentin *

Dipartimento di Scienza dei Materiali, Università degli Studi di Milano-Bicocca, Via Cozzi 55, 20125 Milano, Italy
* Correspondence: cristiana.divalentin@unimib.it

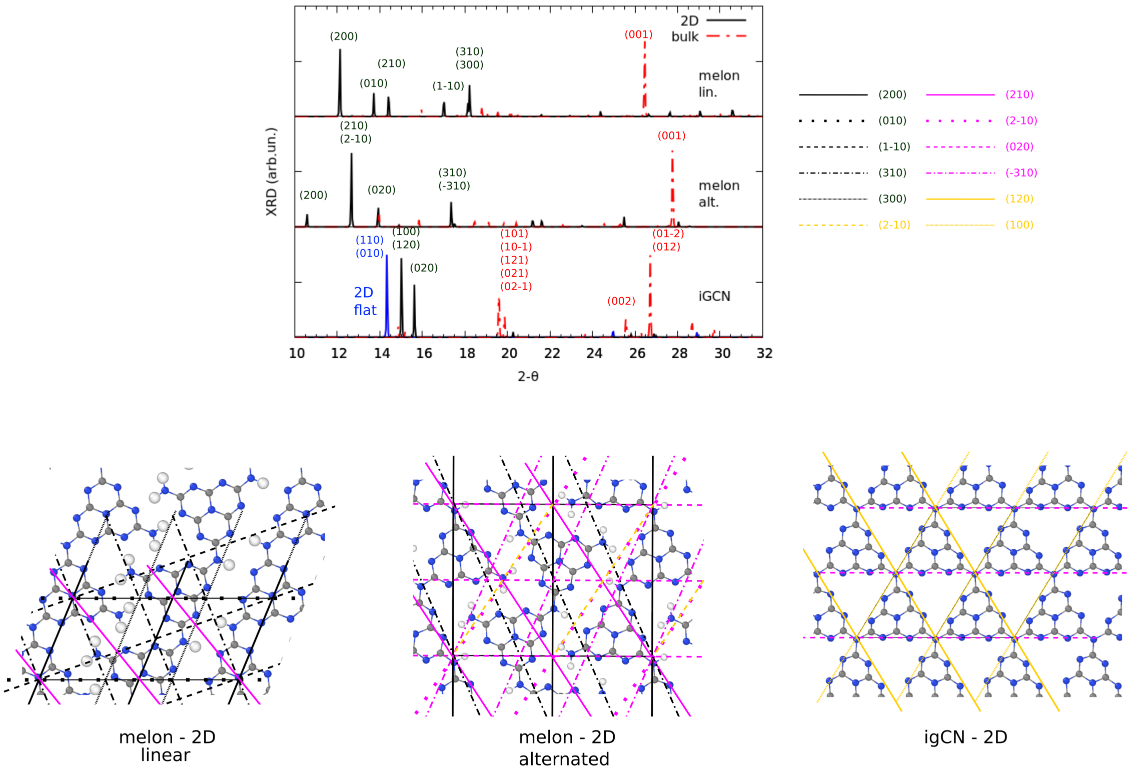

Figure S1. Representation of real space reflection planes associated with the main peaks reported in the XRD spectra of the 2D models.

Table S1. List of the optimized crystal parameters of the optimized unit cell of the periodic models.

Model	**Melon linear**		**Melon alternated**		**igCN**	
Crystal parameter	2D	bulk	2D	bulk	2D	bulk
$\|\mathbf{a}\|$ (Å)	15.80	15.70	16.72	16.67	13.80	13.86
$\|\mathbf{b}\|$ (Å)	6.98	7.00	12.71	12.70	13.31	13.55
$\|\mathbf{c}\|$ (Å)	-	6.93	-	4.66	-	6.96
$\theta(\mathbf{ab})$	67.46°	67.72°	90.03°	90.03°	58.79°	59.25°
$\theta(\mathbf{ac})$	-	100.75°	-	51.55°	-	90.08°
$\theta(\mathbf{bc})$	-	86.37°	-	61.18°	-	90.08°

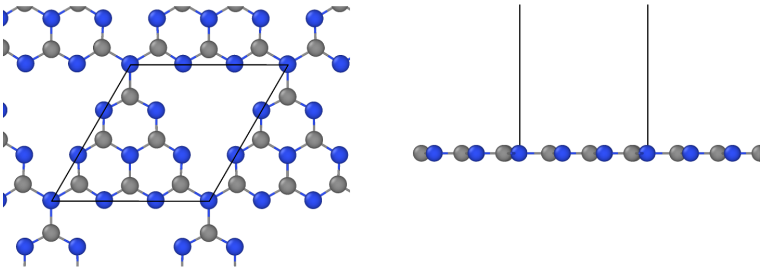

Figure S2. Top and left side view of the optimized structure of the alternative 2D gCN (flat) systems.

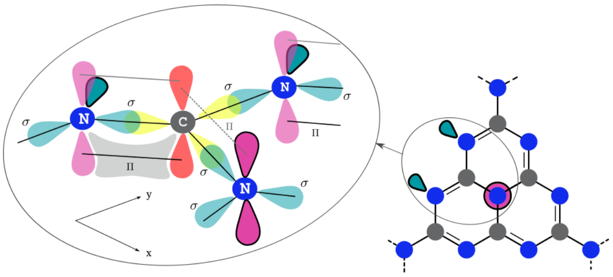

Figure S3. Sketch of a monomer of 2D igCN and its electronic structure in real space. The orbitals where the lone pair should be located are marked by a thicker black border. Red or purple lobes indicate the p_z-like orbitals of C or N, respectively. Yellow/light-blue report $p_{x,y}$-like orbitals on C/N.

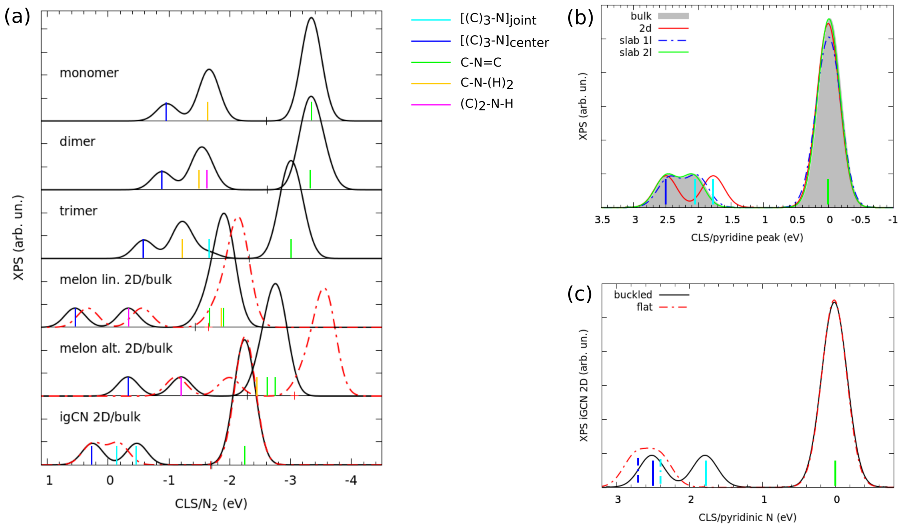

Figure S4. Comparison of simulated XPS spectra (**a**) including the relative chemical shift, calculated with the inclusion of one N_2 molecule in the supercell, (**b**) of bulk/2D/slab igCN and (**c**) 2D igCN flat/distorted, reported using the average total energy of core-excited pyridinic N atoms as reference.

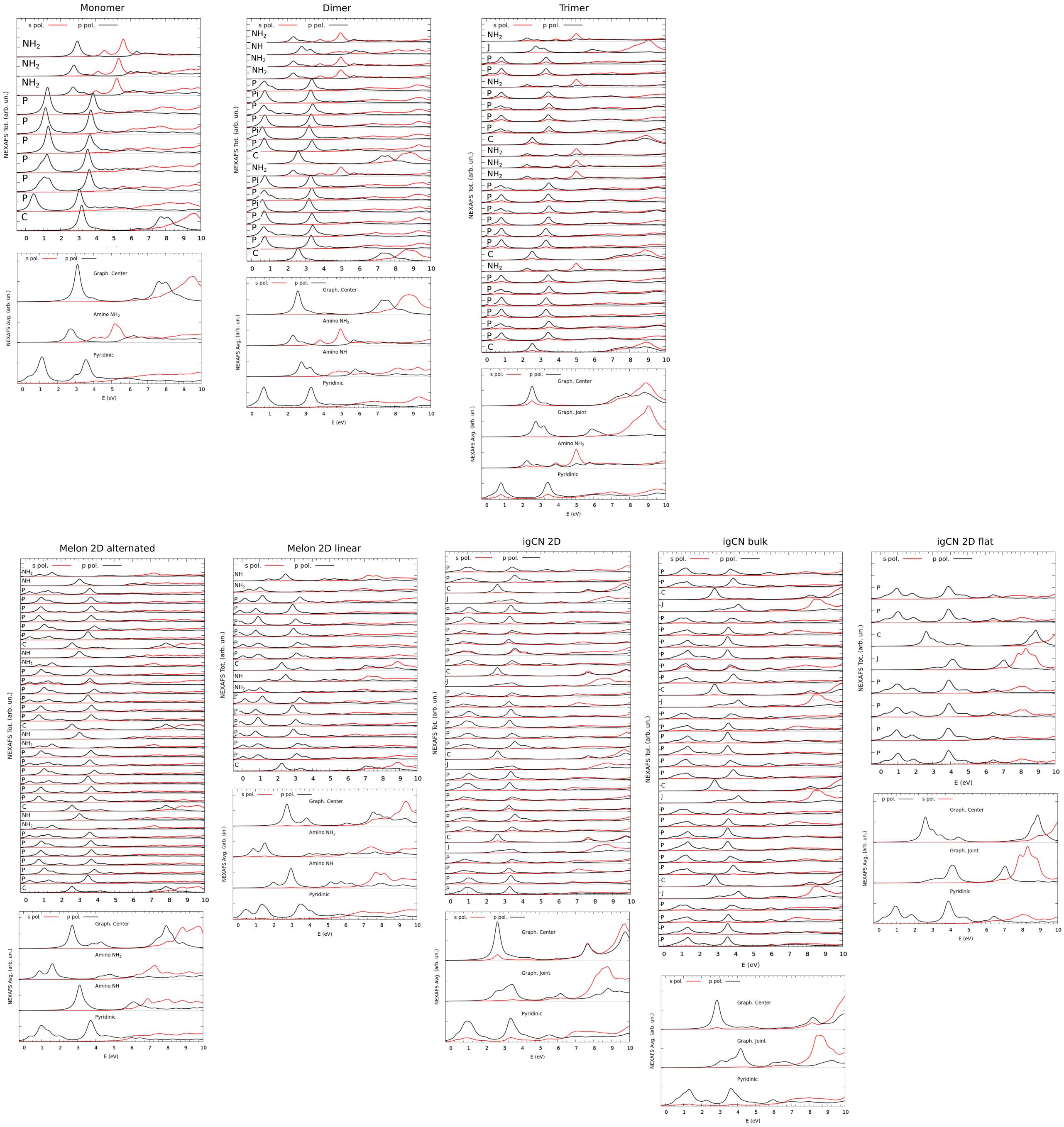

Figure S5. Detailed atomically resolved NEXAFS spectra for the different models, including both in-plane and out-of-plane polarizations. The lower panel show the spectra averaged over the atoms belonging the same N species. All the spectra have been aligned to the onset of the absorption.
